# Prevalence of *Bartonella* species in shelter cats and their ectoparasites in southeastern Brazil

**DOI:** 10.1590/S1984-29612022006

**Published:** 2022-02-21

**Authors:** Juliana Macedo Raimundo, Andresa Guimarães, Gleice Marques Amaro, Aline Tonussi da Silva, Caio Junior Balduino Coutinho Rodrigues, Huarrisson Azevedo Santos, Elba Regina Sampaio de Lemos, Alexsandra Rodrigues de Mendonça Favacho, Cristiane Divan Baldani

**Affiliations:** 1 Laboratório de Patologia Clínica Veterinária, Departamento de Medicina e Cirurgia Veterinária, Instituto de Veterinária, Universidade Federal Rural do Rio de Janeiro – UFRRJ, Seropédica, RJ, Brasil; 2 Laboratório de Controle Microbiano, Departamento de Parasitologia Animal, Instituto de Veterinária, Universidade Federal Rural do Rio de Janeiro – UFRRJ, Seropédica, RJ, Brasil; 3 Laboratório de Sanidade Avícola, Departamento de Epidemiologia e Saúde Pública, Instituto de Veterinária, Universidade Federal Rural do Rio de Janeiro – UFRRJ, Seropédica, RJ, Brasil; 4 Laboratório de Hantaviroses e Rickettsioses, Fundação Oswaldo Cruz – Fiocruz, Rio de Janeiro, RJ, Brasil; 5 Fiocruz Mato Grosso do Sul, Fundação Oswaldo Cruz – Fiocruz, Campo Grande, MS, Brasil

**Keywords:** Bartonella, zoonotic diseases, pathogen transmission, fleas, ticks, lice, Bartonella, zoonoses, transmissão de patógenos, pulgas, carrapatos, piolhos

## Abstract

Feline *Bartonella* can be transmitted to humans through cat scratches or bites, and between cats, by the flea *Ctenocephalides felis*. The study was carried out in order to investigate the occurrence of *Bartonella* DNA in cats living in shelters and their ectoparasites and the relationship between the infection status of cats and ectoparasites they host. *Bartonella* DNA was detected in 47.8% of the cat blood samples, 18.3% of *C. felis* fleas, 13.3% of flea egg pools and 12.5% of lice pools. *B. henselae* and *B. clarridgeiae* DNA were detected in cat fleas, while *B. henselae*, *B. clarridgeiae* and *B. koehlerae* were found in blood samples from bacteremic cats. Cats infested by positive ectoparasites showed approximately twice the odds of being infected. Our results indicate that shelter cats have high prevalence of *Bartonella* species that are known to be human pathogens. This highlights the importance of controlling infestations by ectoparasites to avoid cat and human infection.

## Resumo

Algumas espécies de *Bartonella* têm os felinos como principais hospedeiros reservatórios. Tais patógenos são transmitidos ao homem por intermédio da arranhadura ou mordedura de gatos e entre os gatos, por meio da pulga *Ctenocephalides felis*. O objetivo deste estudo foi investigar a ocorrência de DNA de *Bartonella* spp. em gatos de abrigos e seus ectoparasitas e a relação entre o estado de infecção dos gatos e dos ectoparasitas albergados por estes. Material genético bacteriano foi detectado em 47,8% das amostras de sangue de gatos, 18,3% das pulgas *C. felis*, 13,3% dos "pools" de ovos de pulgas e 12,5% dos "pools" de piolhos. DNA de *B. henselae* e *B. clarridgeiae* foi detectado em pulgas, e *B. henselae*, *B. clarridgeiae* e *B. koehlerae,* em amostras de sangue de gatos. Gatos infestados por ectoparasitas que carreavam DNA de *Bartonella* spp. demonstraram aproximadamente o dobro de chance de estarem infectados. Esses resultados indicam que os gatos de abrigos têm alta prevalência de infecção por espécies de *Bartonella,* capazes de causar doenças no homem. E também destacam a importância do controle e prevenção da infestação por ectoparasitas, no intuito de prevenir a infecção em gatos e humanos.

## Introduction

In the light of One Health concepts, studies on bartonellosis are important because the bacterial genus *Bartonella* can infect a wide variety of animals. This genus is being linked to an ever-increasing number of human diseases that are transmitted by arthropod vectors (Regier et al., 2016). According to the List of Prokaryotic Names with Standing in Nomenclature (LPSN 2021), the genus *Bartonella* contains 37 species, 3 subspecies and 25 with a Candidatus status. The potential domesticated and wild animal reservoirs include cats, dogs, rodents, rabbits, ruminants, sea mammals, wild felines, coyotes, roe-deer (*Capreolus capreolus*), elk *(Cervus elaphus),* jackals *(Canis aureus)*, grey and red foxes (*Urocyon cinereoargenteus* and *Vulpes vulpes*). The list of vectors and potential vectors associated with bacterial transmission includes sandflies, fleas, ticks, lice and mites (Baldani et al., 2014).

To date, natural infections in cats caused by seven *Bartonella* species have been reported: *B. henselae*, *B. clarridgeiae*, *B. koehlerae*, *B. bovis*, *B. quintana, B. vinsonii* subsp. *berkhoffii* and *B. capreoli* (Varanat et al., 2009; Chomel & Kasten, 2010; Gil et al., 2013). Most *Bartonella* species infecting humans are zoonotic, and cats appear to be the primary mammalian reservoir for *B. henselae*, *B. clarridgeiae* and *B. koehlerae* (Boulouis et al., 2005). Feline *Bartonella* can be transmitted to humans through scratches or bites. Transmission between cats most often occurs via the flea *Ctenocephalides felis* (Chomel et al., 1996; Kordick et al., 1999; Guptill, 2012), which is a competent vector of *B. henselae* and a potential vector of *B. clarridgeiae* and *B. koehlerae* (Chomel et al., 1996; Tsai et al., 2011).

In Brazil, there are few studies addressing the occurrence and distribution of *Bartonella* spp. in shelter cats. Therefore, the objective of this study was to investigate the prevalence of *Bartonella* infection in shelter cats and in ectoparasites collected from them, and the relationship between *Bartonella* DNA in cats and their ectoparasites.

## Materials and Methods

### Cat sample

The study protocol was approved by the animal use ethics committee at the Federal Rural University of Rio de Janeiro under procedural number 027/2014.

A survey was carried out in six cat shelters in the metropolitan region of Rio de Janeiro, Brazil, from September 2014 to September 2015 by convenience sampling. After obtaining each shelter owner’s permission, approximately 2 mL of blood was aseptically obtained from cats by means of cephalic phlebotomy. These samples were transferred into sterile tubes containing the anticoagulant ethylenediamine tetraacetic acid, and were maintained at −80 °C until used for molecular analyses.

### Ectoparasite collection and identification

Ectoparasites were retrieved manually from the cats, placed in dry sterile tubes and stored at −20 °C until used. All ectoparasites were morphologically identified to genus or species level based on morphological criteria, through observation under a stereoscopic microscope, in accordance with standard taxonomic keys (Urquhart et al., 1998; Barros-Battesti et al., 2006; Linardi & Santos, 2012).

### Molecular detection

Fleas and ticks were placed individually in separate tubes. Lice were divided into pairs or trios per tube. Some fleas laid eggs inside the tubes: these were used as single pools per flea specimen for DNA extraction, as described in the literature (Horta et al., 2007). Briefly, each flea or tick and each pool of flea eggs or lice was ground up in 40 μl of TE buffer (10 mM Tris-HCl; 0.5 mM EDTA; pH 9.0) in a sterile microtube. The final suspension was boiled at 100 °C for 30 minutes and was then maintained at -20 °C until tested by means of PCR.

Regarding the cats, DNA was extracted from 200 μL of whole blood sample using the Relia Prep^TM^ blood gDNA miniprep system (Promega®), in accordance with the manufacturer’s instructions. For quality assurance, a negative control was processed at the same time as the study samples. It should be highlighted that one of the limitations of the present study was the lack of endogenous gene to verify the presence of amplifiable DNA and check the integrity of the DNA template. As so, negative results should be interpreted with caution.

All the DNA samples were screened for the presence of *Bartonella* spp. 16S-23S rRNA intergenic spacer region (ITS), using the primers 321s and 983as (Maggi & Breitschwerdt, 2005); and for the citrate synthase gene (*glt*A), using the primers BhCS.781p and BhCS.1137n (Norman et al., 1995). Following amplification, the PCR products were subjected to horizontal electrophoresis on 1% agarose gel and were stained with GelRed (Biothium, CA, USA). The positive control consisted of *B. henselae* (Houston strain) cultured in HEp-2 cells. All PCR runs were performed with nuclease-free water (Invitrogen, USA) as the negative control. In order to prevent PCR contamination, the DNA extraction, reaction setup, PCR amplification and electrophoresis were performed in separate rooms.

Amplicons from positive flea and cat samples were randomly selected and purified using the Illustra GFX PCR purification kit (GE Healthcare, Buckinghamshire, England, UK). Purified DNA fragments were subjected to sequence confirmation in an automated sequencer (ABI3730xl, Applied Biosystems, CA, USA). Sense and antisense sequences were analyzed using the DNA Sequence Assembler version 4 software and were compared with those deposited in the GenBank DNA database through using the Basic Local Alignment Search Tool (BLAST, National Center for Biotechnology Information).

A phylogenetic reconstruction was inferred using the maximum likelihood method. Nucleotide substitution models were selected based on Bayesian information criterion (BIC scores) and Tamura three-parameter and Kimura-2 parameter models were used to calculate evolutionary distances. The combination of phylogenetic clusters was assessed using a bootstrap test with 1000 replicates, to test different phylogenetic reconstructions. The phylogenetic evaluation was conducted using the Molecular Evolutionary Genetics Analysis (MEGA) software, version 7.0.18.

### Statistical analysis

The relationship between the *Bartonella* infection status of cats and their fleas was evaluated by means of the chi-square test and association between them was expressed as the odds ratio (OR) at a 95% confidence interval. All the analysis were implemented using the Bioestat 5.0 statistical software.

## Results

A total of 115 fleas, 21 lice and one tick were collected from 46 cats. All flea and lice specimens were identified as adults of *Ctenocephalides felis and Felicola subrostratus*, respectively. The tick specimen was identified as a nymph of *Rhipicephalus sanguineus* sensu lato. All the cats sampled presented ectoparasites on their bodies. On average, approximately three fleas or lice were collected per cat (ranges: 1-9 fleas and 2-4 lice).

Overall, 47.8% (22/46) of the cats tested positive for *Bartonella* DNA according to both the ITS and *gltA* gene tests (Table 1). Sequencing confirmed the presence of *Bartonella henselae*, *Bartonella clarridgeiae* and *Bartonella koehlerae* infection among the blood samples. Cases in which ITS and *glt*A sequences from the same cat corresponded to different feline *Bartonella* species were considered to be coinfections.

**Table 1:** Prevalence of *Bartonella* DNA in cats and their ectoparasites in shelters, Rio de Janeiro, Brazil.

Cats					
DNA samples	Ectoparasites				
	Blood	Flea		Lice*	Ticks
		Adults	Eggs*		
Total of samples	46	115	15	8	1
Total PCR positive no.(%)	22 (47.8)	21 (18.3)	2 (13.3)	1(12.5)	0 (0.0)
*glt*A positive no.(%)	21 (45.7)	18 (15.7)	2 (13.3)	1(12.5)	0 (0.0)
ITS positive no.(%)	12 (26.1)	5 (4.3)	0 (0.0)	0 (0.0)	0 (0.0)

* Number of pools.

*Bartonella* DNA was detected in 18.3% (21/115) of the *C. felis* fleas, of which 15.7% (18/115) were by means of the *glt*A gene and 4.3% (5/115) by the ITS region. Bacterial DNA was amplified from both the ITS and the *glt*A fragments in three flea samples. Among the 15 pools of eggs laid by fleas and the 8 pools of lice, 13.3% and 12.5% showed amplification of the expected *Bartonella* spp. *glt*A gene, respectively. *Bartonella henselae* DNA was detected in cat fleas and their respective eggs, while *Bartonella clarridgeiae* DNA was only identified in adult fleas. No eggs or lice tested positive for the ITS region. No amplification of *Bartonella* DNA was obtained in the *R. sanguineus* (s.l.) nymph. *Bartonella henselae* was the predominant species in both fleas and cats (Table 2).

**Table 2:** *Bartonella* species in cats and their fleas (99 to 100% identity), Rio de Janeiro, Brazil.

Shelter 1	Host cat	Ectoparasites	
		Adult	Eggs
211	*B*. *henselae*	*B*. *henselae*	*B*. *henselae*
208	Negative	*	Negative
76	*	*	Negative
Shelter 2			
175	*B*. *clarridgeiae*	*B*. *henselae*	*B*. *henselae*
171	*B*. *koehlerae*	*	Negative
168	*B*. *henselae*	Negative	-
172	*B*. *henselae*	Negative	Negative
178	*	Negative	Negative
180	Negative	*	Negative
173	Negative	*	Negative
Shelter 3			
205	*B*. *clarridgeiae*	*	-
207	*B*. *clarridgeiae*	*	-
201	*B*. *clarridgeiae + B.henselae*	Negative	-
206	Negative	*B*. *clarridgeiae*	**-**
202	*B*. *clarridgeiae*	Negative	Negative
Shelter 4			
117	*B*. *henselae*	*B*. *henselae*	Negative
116	*B*. *henselae*	*	Negative
Shelter 5			
132	Negative	*B*. *clarridgeiae*	-
131	*B*. *clarridgeiae*	Negative	-
129	*B*. *henselae*	Negative	-
Shelter 6			
193	*B*. *henselae*	Negative	-
191	*	*B*. *henselae*	-
195	*B. henselae*	Negative	**-**
200	*B. henselae*	Negative	-
198	Negative	*	-

* Positive sample, but showing weak bands whose DNA concentration was too low to be sequenced.

At least one *Bartonella* species was detected in fleas in each shelter. Additionally, all bacterial species detected in fleas of each shelter were also identified in at least one of its cats. *Bartonella* spp. was also amplified from fleas belonging to apparently uninfected cats and from infected cats infested by negative fleas. Not all fleas had the same *Bartonella* species as their hosts. Two out of every three fleas collected from infected cats carried the same *Bartonella* species as their cat hosts (Table 2). Whereas 60% (9/15) of the cats infested by positive ectoparasites carried *Bartonella* DNA, the prevalence was only 40% (10/25) in those infested by negative ectoparasites. Although cats infested by positive ectoparasites, especially fleas, had more than twice the odds of being infected, there was no statistical association between the cats’ infection status and parasitism by positive fleas (p-value 0.3685; CI 95% 0.6-8.3).

**Figure 1:**
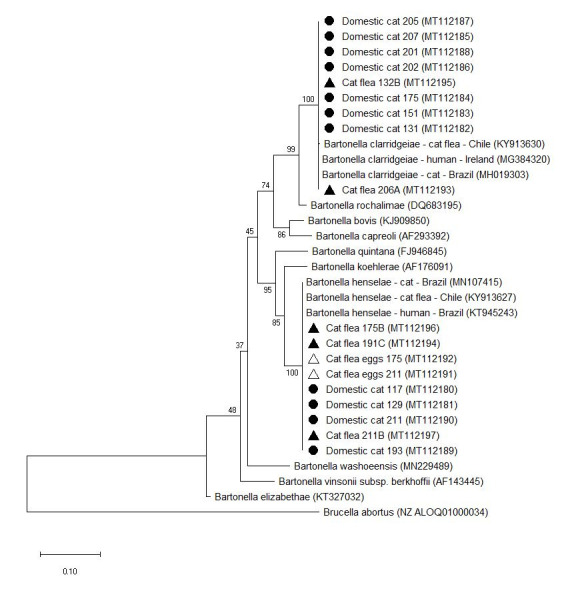
Phylogenetic relationship of *Bartonella species* detected in shelter cats and ectoparasites based on *glt*A gene. Phylogenetic position of *Bartonella* species isolates from shelter cats (●), cat fleas (▲) and cat flea eggs (Δ), Rio de Janeiro, Brazil. The phylogenetic tree was constructed using the maximum likelihood method (K2+G) and the numbers on the tree nodes indicate bootstrap values with 1000 replicates. Accession numbers are indicated. *Brucella abortus* was used as outgroup. The scale bar indicates nucleotide substitutions per site.

**Figure 2:**
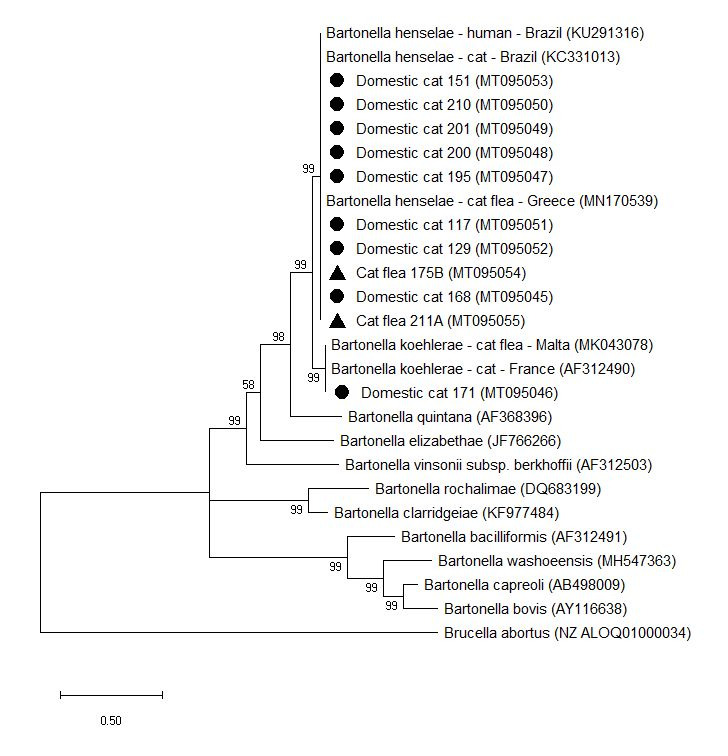
Phylogenetic relationship of *Bartonella* species detected in shelter cats and fleas based on ITS region. Phylogenetic position of *Bartonella* sp. isolates from shelter cats (●) and their fleas (▲), Rio de Janeiro, Brazil. The phylogenetic tree was constructed using the maximum likelihood method (T92+G+I) and the numbers on the tree nodes indicate bootstrap values with 1000 replicates. Accession numbers are indicated. *Brucella abortus* was used as outgroup. The scale bar indicates nucleotide substitutions per site.

All the sequences analysis demonstrated 99 to 100% identity with *B. henselae*, *B. clarridgeiae* and *B. koehlerae* reference sequences (Figures 1 and 2). The phylogenetic tree showed two well-supported clusters (Figure 1) and two clusters contained the human and cat associated *Bartonella* species (Figure 2). The sequences were deposited in GenBank under the accession numbers MT112180 – MT112197 and MT095045 – MT095055 for the *gltA* gene and ITS region, respectively (Figures 1 and 2).

## Discussion

One Health is an initiative that has the aim of bringing together human, animal and environmental health and it plays a significant role in prevention and control of zoonoses (Bidaisee & Macpherson, 2014). The increasingly close health relationship between humans and their domestic animals, especially cats, is conspicuous. According to the Brazilian Association for the Pet Product Industry, the cat population has shown accelerated annual growth in Brazil. Therefore, zoonoses studies have become ever more important. From a public health perspective, cats are a major reservoir host for at least three zoonotic *Bartonella* species (*B. henselae, B. clarridgeiae* and *B. koehlerae*), and commonly infested by *C. felis* fleas. Large flea infestations without proper control can cause even the death of the animal and transmit disease to humans (Limongi et al., 2013) such as plague and bartonellosis (Bitam et al., 2010). To the best of our knowledge, this was the first study in Brazil to investigate *Bartonella* DNA in shelter cats and their ectoparasites.

The overall prevalence of *Bartonella* DNA was 47.5% in cat blood, 18.3% in fleas, 13.3% in flea egg pools and 12.5% in lice pools. *Bartonella* DNA occurred more frequently than previously reported, especially in shelter cats (Bergmans et al., 1997; Alves et al., 2009; Namekata et al., 2010; Staggemeier et al., 2010; Miceli et al., 2013; Braga et al., 2015; Raimundo et al., 2019). However, one previous study reported 97.3% positivity in one shelter in Rio de Janeiro, Brazil (Souza et al., 2010). The risk factors that appear to influence occurrences of bacteremia in cats includes age, flea infestation status, neutering status, historic of fights, outdoor access and multiple-cat households (Chomel et al., 1995; Gurfield et al., 2001; Raimundo et al., 2019; Mazurek et al., 2020). Flea infestation status is particularly important, considering that all the cats in this study had ectoparasites on their body surface. The prevalence of *Bartonella* DNA detected in *C. felis* fleas varies worldwide, ranging from 7.3% to 75.6% (Rolain et al., 2003; Alves et al., 2009; Tsai et al., 2011; Gutiérrez et al., 2015; Rizzo et al., 2015; Furquim et al., 2021). *Bartonella* DNA was not detected in *R. sanguineus* (s.l.) ticks or *F*. *subrostratus* lice collected from shelter cats in Taiwan (Tsai et al., 2011).

This study confirmed the presence of single infections by *B. henselae*, *B. clarridgeiae* and *B. koehlerae*, as well as coinfection by *B. henselae* and *B*. *clarridgeiae*, in feline blood samples. Occurrence of concurrent infection by two or more *Bartonella* species in cats are uncommon in the literature, such that these have either been documented in low percentages of cats or been absent (Gil et al., 2013; André et al., 2016; Gutiérrez et al., 2015; Rizzo et al., 2015). The *Bartonella* species encountered in the present study in cat fleas (*B. henselae* and *B. clarridgeiae*) have also been detected in cat fleas in previous studies (Alves et al., 2009; Assarasakorn et al., 2012; Rojas et al., 2015; Fontalvo et al., 2017).

In all the shelters, *Bartonella* spp. were detected in fleas and their hosts. In one shelter, the *Bartonella* species detected in fleas and their eggs were different from those in their respective host. However, such species have been detected in other cats sharing the same environment. For such non-coincident cases, it is possible that the fleas previously had fed on infected cats other than the one from which they were collected or the bacterial loads in cats’blood samples are at, or below, the detection limit of PCR.

Interestingly, bacterial DNA was detected both in fleas collected from negative hosts and in cats harboring negative fleas. It is noteworthy that in this study, the fleas collected from cats represented a sample of the real flea population present on cats and in the local environment. Thus, newly emerged or not-yet-infected fleas may have been collected. Examples of different bacterial species in fleas and cat hosts have been documented previously (La Scola et al., 2002; Gabriel et al., 2009; Bai et al., 2015; Gutiérrez et al., 2015; Fontalvo et al., 2017).

The presence of *Bartonella* DNA in *C. felis* fleas collected from infected cats also suggested that these ectoparasites play an essential role in the transmission of *Bartonella* species to cats. Studies show that *C. felis* is an important vector for *Bartonella* species, including those for which cats serve as natural reservoir, such as *B. henselae* and *B. clarridgeiae* (Chomel et al., 1996; Bouhsira et al., 2013). In fact, there is a positive correlation between previous or current flea infestation and *Bartonella* molecular positivity in shelter cats (Raimundo et al., 2019). Although there was no statistical association, cats infested by fleas were found to have at least twice the chance of becoming infected by *Bartonella* species. Similarly, previous studies found no apparent correlation (La Scola et al., 2002; Bai et al., 2015).

To the best of our knowledge, the present study provided the first record of detection of *Bartonella* DNA in lice collected from an infected cat. This cat was infested with lice at the time of sample collection, had a history of flea infestation and was living in shelter 6, where *Bartonella* species DNA was also detected in fleas and contact cats. Considering the importance of fleas for *Bartonella* transmission between cats (Chomel et al., 1996; Guptill, 2012; Raimundo et al., 2019), the absence of links between louse infestation and *Bartonella* infection in cats, in this and previous studies (Tsai et al., 2011; Raimundo et al., 2019), and the possibility that lice while feeding on epidermal debris or fur, have ingested the feces of infected fleas, the molecular evidence of *Bartonella* may be accidental and not really responsible for transmission of bacteria to this cat in the present study. Future studies are necessary to evaluate if this arthropod could play a biological role for *Bartonella* transmission among cats.

The possibility of vertical *Bartonella* spp. transmission among fleas remains a possible hypothesis. In our study, *B. henselae* DNA was detected in naturally infected fleas and their eggs. In support of our finding, *Bartonella washoensis and Bartonella vinsonii* subsp. *arupensis* DNA was previously detected in the reproductive tissues (ovaries) of flea species collected from several mammals (*Cynomys ludovicianus, Peromyscus maniculatus* and *Vulpes vulpes*), thus suggesting that transovarian transmission of this organism among fleas may be possible (Brinkerhoff et al., 2010). On the other hand, in another study, no *Bartonella* DNA was amplified in eggs laid by infected fleas at experimental condition, and authors concluded that those results could not be extended to natural conditions (Bouhsira et al., 2013). Knowledge of *Bartonella* behavior and dispersal in fleas is limited, and the question of whether fleas can acquire *Bartonella* by means of mechanisms other than ingestion of infected blood remains to be answered. According to a study evaluating ticks as a possible vector of *B. henselae*, transovarian transmission was not confirmed as bacterial DNA was detected in eggs laid by females fed on blood containing *B. henselae* but not in larvae obtained from these eggs (Cotté et al., 2008). Those findings suggest the external contamination of eggs with infected flea feces and for this reason, further studies are needed to investigate the vertical transmission hypothesis.

This study showed that three distinct *Bartonella* species (*B. henselae*, *B. clarridgeiae* and *B. koehlerae*) occur in shelter cats in the metropolitan region of Rio de Janeiro and that *B. henselae* and *B. clarridgeiae* circulate among fleas collected from them, thus emphasizing the importance of this ectoparasite in bacterial transmission between cats. For public health purposes, it is important to emphasize the relationship between the *Bartonella* species identified in ectoparasites and their cats hosts since they are agents associated with human disease. Thus, ectoparasite control measures should be implemented to prevent flea infestation and, consequently, *Bartonella* infection in cats and the humans with whom they have close contact.
